# Cardiac output calculation using the Liljestrand and Zander formula: is this method applicable during immediate transition after birth? — A post hoc analysis

**DOI:** 10.1007/s00431-024-05592-6

**Published:** 2024-05-08

**Authors:** Daniel Pfurtscheller, Bernhard Schwaberger, Nina Höller, Nariae Baik-Schneditz, Lukas Schober, Marlies Bruckner, Christoph Schlatzer, Berndt Urlesberger, Gerhard Pichler

**Affiliations:** 1 Division of Neonatology, Department of Pediatrics and Adolescent Medicine, Medical University of Graz, Auenbruggerplatz 34/2, 8036 Graz, Austria; 2 Research Unit for Neonatal Micro- and Macrocirculation, Department of Pediatrics and Adolescent Medicine, Medical University of Graz, Graz, Austria; 3 Research Unit for Cerebral Development and Oximetry, Department of Pediatrics and Adolescent Medicine, Medical University of Graz, Graz, Austria

**Keywords:** Cardiac output, Neonate, Transition period, Liljestrand and Zander, NICOM

## Abstract

The transition from intrauterine to extrauterine life is a critical period for neonates. Assessing the cardiovascular transition during this period immediately after birth is crucial but challenging. The present study compares adjusted estimated cardiac output values calculated by the Liljestrand and Zander formula (COest/adj LaZ) with non-invasively measured cardiac output values (CO-bioimpedance) during immediate transition after birth. We performed a secondary outcome analysis of a prospective observational study in preterm and term neonates. Ten and 15 min after birth, arterial blood pressure and heart rate were assessed, and CO-bioimpedance was measured using electrical bioimpedance method (Aesculon monitor, Osypka, Germany). We calculated COest/adj LaZ and compared it to CO-bioimpedance. Further, we performed a correlation analysis. Thirty-two neonates with a median (IQR) gestational age of 37.0 (32.0–39.4) weeks were included. Mean ± SD CO-bioimpedance was 0.62 ± 0.15 l/min, and COest/adj LaZ was calculated to be 0.64 ± 0.10 l/min, whereby both correlated significantly (*p* = 0.025, *r* = 0.359) with each other.

*  Conclusion*: The present study demonstrates high comparability of COest/adj LaZ and CO-bioimpedance in neonates during immediate transition after birth, suggesting that cardiac output can be derived in a cost-effective and feasible manner if other methods are not available.

**Table:** 

**What is Known:** *• Echocardiography is considered the gold standard for non-invasive CO evaluation, but its feasibility during the immediate transition period is limited.*	
**What is New:** *• Non-invasive methods such as CO-bioimpedance for cardiac output (CO) measurement and the Liljestrand and Zander (LaZ) formula for estimating CO offer promising alternatives during the immediate transition period.*

**What is Known:**

*• Echocardiography is considered the gold standard for non-invasive CO evaluation, but its feasibility during the immediate transition period is limited.*

**What is New:**

*• Non-invasive methods such as CO-bioimpedance for cardiac output (CO) measurement and the Liljestrand and Zander (LaZ) formula for estimating CO offer promising alternatives during the immediate transition period.*

## Introduction

The immediate neonatal transition after birth is associated with complex cardio-circulatory changes and is one of the most vulnerable periods in life [1]. Consequently, the clinical evaluation of the neonate’s condition after birth is crucial and consistently includes Apgar scoring. The Apgar score is designed to assess for signs of hemodynamic compromise. However, due to its inter-observer variability and the limitations regarding preterm neonates, its reliability is debatable [2, 3]. Recent resuscitation guidelines recommend the use of pulse oximetry and electrocardiogram (ECG) for initial assessment of neonates to identify those in need for medical support [4, 5]. The routinely monitored heart rate (HR) alone may not always reflect failure of cardio-circulatory transition sufficiently. Assessment of arterial blood pressure might provide additional information. Even though our research group established reference ranges for arterial blood pressure during this period after birth, its value is not clear yet [6, 7]. For a more thorough understanding of cardio-circulatory changes during neonatal transition, cardiac output (CO) assessment might add important information. The gold standard in terms of non-invasive evaluation of CO is echocardiography, which, however, is not always feasible during this immediate transition period [8]. Non-invasive CO measurement (CO-bioimpedance) using electrical bioimpedance emerges as an alternative; hence, its practicability within the initial minutes after birth poses challenges but its accuracy appears acceptable [9, 10]. An alternative tool to gain information on CO is the Liljestrand and Zander (LaZ) formula [11] which has been described to be one of the most adequate pulse pressure wave analysis (PPWA) [12]. The application of this formula to estimate CO might be a promising alternative to other more complex methods of CO assessment during the immediate transition in neonates.

The aim of this study was to assess the adjusted estimated CO values calculated by the LaZ formula (COest/adj LaZ) and compare these with CO values non-invasively measured by electrical bioimpedance (CO-bioimpedance) during immediate transition in preterm and term neonates.

## Materials and methods

### Design

The present study is a post hoc analysis of secondary outcome parameters of a prospective observational study performed at the Division of Neonatology, Department of Pediatrics and Adolescent Medicine, at the Medical University of Graz, Austria (EC number: 30–450 ex 17/18).

Written parental consent was obtained before birth for all neonates included in the prospective observational study. Demographics and antepartum medical history of neonates were collected from patient charts.

### Inclusion and exclusion criteria

Preterm and term neonates delivered by caesarean section due to feasibility to get informed consent before birth were included in the prospective observational study and were eligible for the present post hoc analysis. Neonates with ECG monitoring, arterial blood pressure measurements, and electrical bioimpedance-based CO measurements at 10 and 15 min after birth were included for analyses.

Exclusion criteria were major congenital malformations and umbilical cord artery pH < 7.0.

### Monitoring

All neonates were transferred to the resuscitation table immediately after birth and routine monitoring with pulse oximetry and ECG (Intelli Vue MP 30 Monitor, Philips, Amsterdam, The Netherlands) was initiated.

HR was continuously obtained by ECG during the first 15 min after birth. Arterial blood pressure was measured non-invasively at minutes 10 and 15 after birth using an oscillometric blood pressure cuff (Intelli Vue MP 30 Monitor, Philips, Amsterdam, The Netherlands) of appropriate size (#1, #2, or #3) on the right calf. The cuff size diameter was chosen according to the circumference of the infant’s right calf. The right calf was chosen to avoid interference with the pulse oximetry measurements at the infant’s right hand or wrist.

### CO-bioimpedance measurement

For measurements of CO-bioimpedance, the Aesculon monitor (Osypka, Berlin, Germany) was used. Before starting the measurement, the skin was cleaned and four electrodes were placed on the left forehead, left side of the neck, left hemithorax, and left thigh. The CO measurements at minutes 10 and 15 after birth were only accepted if the Signal Quality Index (SQI) was ≥ 80%.

All data were continuously stored in a polygraphic system (alpha trace digital MM, BEST Medical Systems, Vienna, Austria) for further analyses.

### COest/adj LaZ calculation

Estimated CO (COest) was calculated using the LaZ formula:

$$\mathrm{COest }=\mathrm{ PP }/ (\mathrm{SABP }+\mathrm{ DABP}) *\mathrm{HR }(\mathrm{no\;unit})$$

(PP: pulse pressure, SABP: systolic arterial blood pressure, DABP: diastolic arterial blood pressure, HR: heart rate).

CO was then adapted with the calibration factor (*k*) to gain the adjusted estimated CO (COest/adj LaZ).

$${\text{COest}}/\mathrm{adj\;LaZ }= k* (\mathrm{PP }/ (\mathrm{SABP }+\mathrm{ DABP}) *{\text{HR}}) ({\text{unit}}:\mathrm{ l}/{\text{min}})$$

### Statistical analysis

Demographic and clinical data are presented as mean ± standard deviation (SD) for normally distributed data or as median and interquartile range (IQR) for skewed distributions.

Correlation analysis of CO-bioimpedance and COest/adj LaZ was performed using Pearson’s correlation, due to this data being normally distributed. The Kolmogorov–Smirnov test was performed prior to establishing normality. In addition, the Bland–Altman analysis was performed.

A *p*-value < 0.05 was considered statistically significant. These values were considered in an explorative sense so that no multiple testing corrections were performed. All statistical analyses were performed using IBM SPSS Statistics 28 (IBM Corporation, Armonk, NY, USA).

## Results

Between December 2018 and November 2019, 80 preterm and term neonates were included in the prospective observational study and eligible for analysis. Forty-eight neonates were excluded because CO-bioimpedance data showed a SQI < 80%. No additional neonates were excluded for any other reasons.

The demographic and clinical data presented in Table 1 are within normal ranges. Within the cohort, there were two neonates classified as small for gestational age (SGA). Additionally, the favorable Apgar scores and arterial pH values indicate stability among the newborn neonates.

**Table 1:** Demographic and clinical data for term and preterm neonates

Gestational age, weeks	37.0 (32.0–39.4)		
Birth weight, g	2801 (1510–3800)		
Female *n* (%)	18 (56)		
Umbilical artery pH	7.31 ± 0.04		
Apgar 1 min	9 (7–9)		
Apgar 5 min	10 (9–10)		
Apgar 10 min	10 (9–10)		
COest (no unit)	41.22 ± 14.66		
	CO-bioimpedance (*n* = 32)	Calculated COest/adj LaZ (*n* = 32)	*p*-value
CO l/min	0.62 ± 0.15	0.64 ± 0.10	0.472
Correlation	CO-bioimpedance with calculated COest/adj LaZ (*n* = 32)	CO-bioimpedance with calculated COest (*n* = 32)	
*p*	0.025*	0.025*	
*r*	0.359	0.359	

Data are presented as mean ± SD, median (IQR), or *n* (%)

*CO* cardiac output, *est* estimated, *LaZ* Liljestrand and Zander, *CO-bioimpedance* cardiac output gained non-invasively with bioimpedance

*p-values indicate a significant difference

No significant difference was observed between the two CO measurement methods (Table 1).

A correlation analysis of CO-bioimpedance and COest/adj LaZ revealed a significant positive correlation between the CO values (Table 1).

**Fig. 1:**
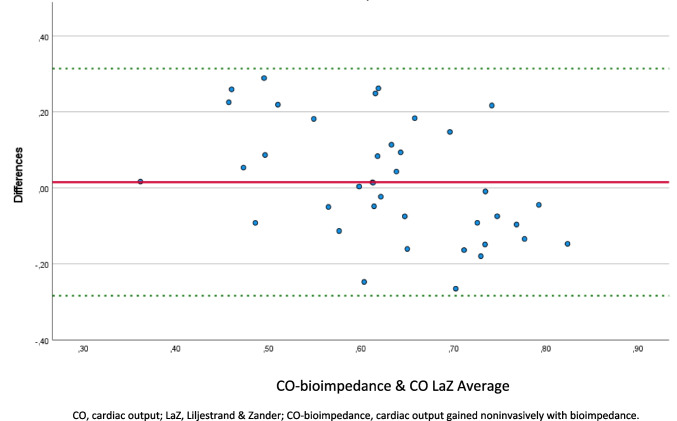
Bland–Altman plot of CO measurements: CO-bioimpedance vs. Liljestrand and Zander formula

In the Bland–Altman plot (Fig. 1), the mean difference between CO-bioimpedance and COest/adj LaZ was 0.015, with a standard deviation (SD) of 0.15, respectively. The upper limit was 0.31 and a lower limit −0.28.

## Discussion

The present study demonstrates comparable results between COest/adj LaZ and CO-bioimpedance during the immediate transition period, indicating that the COest/adj LaZ may be a proper alternative with an acceptable precision. The results are also in accordance with previous findings after the immediate transition [12].

Before comparison of CO-bioimpedance with the COest/adj LaZ, the COest LaZ estimated value underwent adjustment through multiplication by a calibration factor (*k*) as proposed by König and Sun [13]. This adjustment resulting in COest/adj LaZ improved in accordance with the CO-bioimpedance.

Currently, CO-bioimpedance stands as the sole non-invasive method for continuous CO monitoring, demonstrating acceptable precision. However, its main challenge lies in signal quality. Noori et al. compared echocardiography with CO-bioimpedance and described comparable accuracy and precision in CO measurements when the SQI of CO-bioimpedance was equal to or exceeds 80% [14]. Considering this, CO-bioimpedance encounters difficulties, especially during the immediate transition period due to low SQI caused by movements and manipulations, as highlighted in several studies [9, 10]. During the immediate transition, therefore, calculating COest/adj using the LaZ method may prove beneficial for clinicians and researchers, since obtaining COest/adj LaZ based on blood pressure and heart rate with satisfactory quality appears more feasible than with CO-bioimpedance measurement. For instance, in our study, approximately 60% of neonates were excluded due to poor SQI of CO-bioimpedance which is consistent with other studies [9]. In contrast, no neonate was excluded due to missing blood pressure or heart rate values. Despite the high exclusion rate due to the high SQI may be debated, this stringent standard of data quality of CO-bioimpedance bolsters the precision of the comparison of CO-bioimpedance with COest/adj LaZ in the present study.

Besides that, Boet et al. [15] demonstrated a tendency for CO-bioimpedance to overestimate echocardiography readings when CO exceeds 0.4 l per minute in preterm neonates with a mean gestational age of 31 weeks. Our study, however, included neonates with the mean gestational age of 37 weeks that makes comparison to the data of Boet et al. [15] difficult and might explain differences in findings. In contrast, the study conducted by Noori et al. [14] demonstrated in a comparable cohort in neonates with a mean gestational age of 39.2 weeks that CO-bioimpedance readings align closely with echocardiography even for CO values exceeding 0.4 l per minute. Furthermore, it has been noted that the precision of CO-bioimpedance remains uncertain when applied to sick neonates [10]. However, in our cohort, the included neonates after caesarean section did not exhibit severe illness, suggesting further reliability in the CO-bioimpedance measurements and allowing comparison with COest/adj LaZ.

The high comparability between COest/adj LaZ and another CO measurement method is in accordance with PPWA trial by Sun et al. which demonstrated that the LaZ calculation emerged as the most suitable PPWA method [12]. Furthermore, Koenig et al. showed commendable precision of COest/adj LaZ when comparing it to CO derived from the Finometer in adults [13]. The Finometer, a Modelflow device used for CO estimation, has consistently shown, in various adult studies, values akin to those obtained through thermodilution — a more invasive technique considered the gold standard for CO measurement [16].

### Strengths and limitations

A strength of the study is the large sample size included in this study during the immediate transition with reliable CO-bioimpedance data. A limitation is that CO LaZ was not compared to the gold standard (thermodilution) but was compared with CO-bioimpedance, a method that is not considered the gold standard. However, to perform the gold standard (thermodilution) is not feasible during the immediate transition and the precision of CO-bioimpedance is considered acceptable [10]. Another limitation is that no very low gestational age preterm neonates were included. Therefore, differences between both methods cannot be ruled out with the present study.

## Conclusion

In our study, we explored a straightforward formula for estimating CO. Our results indicate that calculated COest/adj using the LaZ formula was comparable and correlated significantly with CO-bioimpedance in healthy neonates during immediate neonatal transition after birth. These findings have significant clinical implications suggesting that CO can easily be derived in a cost-effective and feasible manner, particularly when other methods are not available.

## Data Availability

The dataset generated and analyzed during the current study is not publicly available due to concerns regarding the privacy of study participants. However, it can be made available from the corresponding author upon reasonable request. Requests for data access should be directed to the corresponding author.
